# Sesamin ameliorates oxidative stress and mortality in kainic acid-induced status epilepticus by inhibition of MAPK and COX-2 activation

**DOI:** 10.1186/1742-2094-8-57

**Published:** 2011-05-24

**Authors:** Peiyuan F Hsieh, Chien-Wei Hou, Pei-Wun Yao, Szu-Pei Wu, Yu-Fen Peng, Mei-Lin Shen, Ching-Huei Lin, Ya-Yun Chao, Ming-Hong Chang, Kee-Ching Jeng

**Affiliations:** 1 Division of Neurology, Taichung Veterans General Hospital, Taichung, Taiwan; 2 Graduate Institute of Biomedicine and Biomedical Technology, National Chi Nan University, Nantou, Taiwan; 3 Department of Biotechnology, Yuanpei University, Hsinchu, Taiwan; 4 Department of Physical Education Office, Yuanpei University, Hsinchu, Taiwan; 5 Department of Medical Research, Taichung Veterans General Hospital, Taichung, Taiwan

**Keywords:** Status epilepticus, PC12 cells, BV-2 cells, sesamin, kainic acid, reactive oxygen species, thiobarbituric acid reactive substances, nitric acid, superoxide dismutase, mitogen-activated protein kinases, COX-2

## Abstract

**Background:**

Kainic acid (KA)-induced status epilepticus (SE) was involved with release of free radicals. Sesamin is a well-known antioxidant from sesame seeds and it scavenges free radicals in several brain injury models. However the neuroprotective mechanism of sesamin to KA-induced seizure has not been studied.

**Methods:**

Rodents (male FVB mice and Sprague-Dawley rats) were fed with sesamin extract (90% of sesamin and 10% sesamolin), 15 mg/kg or 30 mg/kg, for 3 days before KA subcutaneous injection. The effect of sesamin on KA-induced cell injury was also investigated on several cellular pathways including neuronal plasticity (RhoA), neurodegeneration (Caspase-3), and inflammation (COX-2) in PC12 cells and microglial BV-2 cells.

**Results:**

Treatment with sesamin extract (30 mg/kg) significantly increased plasma α-tocopherol level 50% and 55.8% from rats without and with KA treatment, respectively. It also decreased malondialdehyde (MDA) from 145% to 117% (*p *= 0.017) and preserved superoxide dismutase from 55% of the vehicle control mice to 81% of sesamin-treated mice, respectively to the normal levels (*p *= 0.013). The treatment significantly decreased the mortality from 22% to 0% in rats. Sesamin was effective to protect PC12 cells and BV-2 cells from KA-injury in a dose-dependent manner. It decreased the release of Ca^2+^, reactive oxygen species, and MDA from PC12 cells. Western blot analysis revealed that sesamin significantly reduced ERK1/2, p38 mitogen-activated protein kinases, Caspase-3, and COX-2 expression in both cells and RhoA expression in BV-2 cells. Furthermore, Sesamin was able to reduce PGE_2 _production from both cells under KA-stimulation.

**Conclusions:**

Taken together, it suggests that sesamin could protect KA-induced brain injury through anti-inflammatory and partially antioxidative mechanisms.

## Background

Status epilepticus (SE) is defined as a period of continuous seizure activity [1,2]. Prolonged febrile seizures and SE have been implicated as a major predisposing factor for the development of mesial temporal sclerosis and temporal lobe epilepsy [1,3]. This emergency condition requires a prompt and appropriate treatment to prevent brain damage and eventual death. In animal models, similar pathologic changes can be observed with electrically and chemically induced seizures [4-7]. Animal studies show that SE causes recurrent spontaneous seizures (epilepsy) [6,8,9] and releases free radicals from experimental models of kainic acid (KA), pilocarpine, pentylenetetrazole, and ferric chloride [10-14].

KA, a glutamate related chemical, induces neuronal excitability, reactive oxygen species (ROS) production and lipid peroxidation in neurons [15-17]. It triggers neuronal membrane depolarization by the release of calcium ions which are involved in nerve impulse transmission as the calcium action potential reaches the synapse [15]. The apoptosis of nerve cells can be triggered by a large number of intracellular calcium influx [18]. Mitogen-activated protein kinases (MAPKs) and Rho kinases are also associated with seizures, inflammation and apoptosis [19-21].

Sesamin and sesamolin are the major lignans from sesame seeds. Previously, we and others report that sesamin can protect against hypoxia-, H_2 _O_2 _-or 1-methyl-4-phenyl-pyridine (MPP^+^)-induced brain and PC12 cells injuries [22,23]. Various plant antioxidants have been shown to protect brain form KA-induced calcium ions and ROS [24-26]. Sesamin also inhibits nitric oxide (NO) and cytokine production in lipopolysaccharide (LPS)-and oxidative-stressed BV-2 microglia [27,28].

It is perceivable that sesamin could protect animal from KA-induced SE as in other brain injury models. Therefore, this study investigated the effect of sesamin on the KA-induced SE animals as well as the protective mechanism in neuronal PC12 cells and microglial BV-2 cells.

## Methods

### Reagents

Pure sesamin and sesamin extract (90% sesamin and 10% sesamolin, as determined by HPLC) were purchased from Joben Bio-medical co. (Kaohsiung, Taiwan). Kainic acid (KA) was obtained from Sigma-Aldrich (Steinheim, Germany) and Cayman Chemical (Ann Arbor, MI, USA), 2', 7'-dichlorodihydrofluorescein diacetate (H_2 _DCF-DA) was obtained from Molecular Probes (Eugene, OR, USA).

### Oxidative Stress in mice

Adult male FVB mice, 23-25 g of weight, were used for the study. SE was induced with KA (30 mg/ml in phosphate-buffered saline (PBS), 30 mg/kg, s.c.). Sesamin extract was diluted with corn oil (30 mg/ml). The animals were fed with 2 different dosages of sesamin extract (15 mg/kg or 30 mg/kg) by gavage for 3 days before the KA experiment. The vehicle control group was fed with equal volume of corn oil. The procedures were approved by the Institutional Animal Care and Use Committee, Taichung Veterans General Hospital (IACUC Approval No. LA-97490) and all possible steps were taken to avoid animals' suffering at each stage of experiments.

Diazepam at lethal dosage, 60 mg/kg i.p., was given to stop seizures 2 h after KA injection and the animal was sacrificed by decapitation under CO_2 _asphyxia. The naïve animal serves as a control. The whole brain was removed and immediately frozen in liquid nitrogen and stored at-70°C until use.

Malondialdehyde (MDA) as a part of thiobarbituric acid reacting substances (TBARS) was used as an indicator of lipid peroxidation. To estimate oxidative stress, the amount of TBARS in the brain from each group was measured. Manual homogenization of brains was carried out at 4°C with a cold buffer. Protein concentration of the homogenate was determined by BCA protein assay using bovine serum albumin as a standard. The detection of TBARS was from the modified method of Ohkawa [29]. Briefly, the sample (0.2 ml) was mixed with the same volume of 20% (w/v) trichloroacetic acid and 1% (w/v) thiobarbituric acid in 0.3% (w/v) NaOH. The mixture was heated in the water bath at 95°C for 40 min, cooled to room temperature and centrifugation at 5000 rpm for 5 min at 4°C. The fluorescence of the supernatant was determined by a spectrophotometer with excitation at 544 nm and emission at 590 nm.

Superoxide dismutase (SOD) activity was determined by a RANSOD kit (Randox, USA). This method was based on the formation of red formazan from the reaction of 2-(4-iodophenyl)-3-(4-nitrophenol)-5-phenyltetrazolium chloride and superoxide radical and assayed in a spectrophotometer at 505 nm. The inhibition of the produced chromogen was proportional to the activity of the SOD present in the sample. A 50% inhibition was defined as one unit of SOD, and specific activity was expressed as units per milligram protein.

### Measurement of tocopherols

Plasma α-tocopherol was determined by HPLC method. Briefly, plasma was treated with anti-oxidant reagent (0.25% BHT and 0.2% vitamin C in methanol, 1:7), mixed and centrifuged for 10 min × 12000 *g*. Sample (20 μl) or internal control was then injected to HPLC system (BAS PM-80) with C18 column (4.6 × 150 mm, 5 μm; mobile phase, 95% methanol, flow rate: 1.0 ml/min) and fluorescence detector with 296 nm excitation, and 340 nm emission.

### Mortality and behavior

Adult male Sprague-Dawley rats weighing 380-420 g were used to study the protective effect of sesamin extract. Rats were fed with sesamin extract (30 mg/kg/day) for 3 days before the SE experiment. The control group was treated with the vehicle corn oil. SE was induced with kainic acid (12 mg/kg, in PBS, s.c.). Each behavioral seizure was recorded according to a modified classification from Racine [30]: 0, immobility or exploring; 1, facial clonus; 2, head nodding; 3, unilateral forelimb clonus; 4, bilateral forelimb clonus and rearing; 5, falling; 6, repeated falling; 7, bouncing; 8, generalized tonus. Four behavioral patterns of SE could be recognized: immobile (class 0), exploratory (class 0), masticatory (class 1-2) and clonic (class 3-8) [31]. Diazepam, 25 mg/kg i.p., was given to stop seizures at 5 h of SE and the 10-h mortality rate was recorded.

### Cell culture

Rat pheochromacytoma (PC12) cells and murine microglial BV-2 cells were maintained in Dulbecco's modified Eagle's medium (DMEM) supplemented with 10% fetal bovine serum (FBS), 5% heat-inactivated horse serum, 100 U/ml penicillin and 100 μg/ml streptomycin at 37°C in a humidified incubator under 5% CO_2_. Confluent cultures were passaged by trypsinization. For experiments, cells were washed twice with warm DMEM (without phenol red), then treated in serum-free medium. In all experiments, cells were treated with and without sesamin and/or KA-stress for the indicated times.

### Cell viability

Cell viability was measured with blue formazan that was metabolized from colorless 3-(4,5-dimethyl-thiazol-2-yl)-2,5-diphenyl tetrazolium bromide (MTT) by mitochondrial dehydrogenases, which are active only in live cells. PC12 cells were pre-incubated in 24-well plates at a density of 5 × 10^5 ^cells per well for 24 h, and then washed with PBS. Cells with various concentrations of sesamin were treated with 150 mM KA for 24 h, and grown in 0.5 mg/ml MTT at 37°C. One hour later, 200 μl of solubilization solution was added to each well and absorption values read at 540 nm on an automated spectraMAX 340 (Molecular Devices, Sunnyvale, CA, USA) microtiter plate reader. Data were expressed as the mean percent of viable cells vs. control.

### Lactate dehydrogenase (LDH) release assay

Cytotoxicity was determined by measuring the release of LDH. PC12 cells or BV-2 cells with various concentrations of sesamin were treated with 150 mM KA for 24 h and the supernatant was used to assay LDH activity. The reaction was initiated by mixing 0.1 ml of cell-free supernatant with potassium phosphate buffer containing nicotinamide adenine dinucleotide (NADH) and sodium pyruvate in a final volume of 0.2 ml to 96-well plate. The rate of absorbance was read at 490/630 nm on a spectraMAX 340 instrument. Data were expressed as the mean percent of viable cells vs. 150 mM KA control.

### Calcium release

PC12 cells or BV-2 cells with various concentrations of sesamin were treated with 150 mM KA for 24 h and the supernatant was used to assay the release of Ca^2+^. The 10 μl supernatant was added to 1 ml Ca^2+ ^reagent (Diagnostic Systems, Holzheim, Germany) and mixed well, stood for 5 min, then transferred the 100 μl aliquot to 96 well. The calcium concentration was determined using a microplate reader with a 620 nm absorbance and quantified with a 10 mg/ml Ca^2+ ^standard solution.

### Reactive oxygen species generation

Intracellular accumulation of ROS was determined with H_2 _DCF-DA. This nonfluorescent compound accumulates within cells upon deacetylation. H_2 _DCF then reacts with ROS to form fluorescent dichlorofluorescein (DCF). PC12 cells or BV-2 cells were plated in 96-well plates and grown for 24 h before addition of DMEM plus 10 μM H_2 _DCF-DA, incubation for 60 min at 37°C, and treatment with 150 μM KA for 60 or 120 min. Cells were then washed twice with room temperature Hank's balanced salt solution (HBSS without phenol red). Cellular fluorescence was monitored on a Fluoroskan Ascent fluorometer (Labsystems Oy, Helsinki, Finland) using an excitation wavelength of 485 nm and emission wavelength of 538 nm.

### Measurement of lipid peroxidation

Lipid peroxidation is quantified by measuring malondialdehyde (MDA) of PC12 cells by lipid peroxidation (LPO) assay kit (Cayman Chemical, Ann Arbor, MI, USA). This kit works on the principle of condensation of one molecule of either MDA or 4-hydroxyalkenals with two molecules of N-methyl-2-phenylindole to yield a stable chromophore. MDA levels were assayed by measuring the amount expressed in 5 × 10^5 ^cells and the absorbance at 500 nm was determined using an ELISA reader (spectraMAX 340).

### Preparation of cell extracts

Test medium was removed from culture dishes and cells were washed twice with ice-cold PBS, scraped off with a rubber policeman, and centrifuged at 200 × *g *for 10 min at 4°C. The cell pellets were resuspended in an appropriate volume (~4 × 10^7 ^cells/ml) of lysis buffer containing 20 mM Tris-HCl, pH 7.5, 137 mM NaCl, 1 mM phenylmethylsulfonylfluoride, 10 μg/ml aprotinin, 10 μg/ml leupeptin, and 5 μg/ml pepstain A. The suspension was then sonicated. Protein concentration of samples was determined by Bradford assay (Bio-Rad, Hemel, Hempstead, UK) and samples equilibrated to 2 mg/ml with lysis buffer.

### Western blotting

Protein samples from PC12 cells or BV-2 cells containing 50 μg of protein were separated on 12% sodium dodecyl sulfate-polyacrylamide gels and transferred to immobile polyvinylidene difluoride membranes (Millipore, Bedford, MA). Membranes were incubated for 1 h with 5% dry skim milk in TBST buffer (0.1 M Tris-HCl, pH 7.4, 0.9% NaCl, 0.1% Tween-20) to block nonspecific binding, and then incubated with rabbit anti-COX-2, Rho A (1:1000; Cayman chemical; Cell Signaling, USA), and anti-phospho-MAPKs. Subsequently, membranes were incubated with secondary antibody streptavidin-horseradish peroxidase conjugated affinity goat anti-rabbit IgG (Jackson, West Grove, PA, USA).

### Statistical analysis

Data were expressed as the mean ± SD and mean ± SE, in vivo and in vitro experiments, respectively. For single variable comparisons, Student's test was used. For multiple variable comparisons, data were analyzed by one-way analysis of variance (ANOVA) followed by Scheffe's test or the least significant differences post-hoc test. Categorical variables were analyzed by use of Fisher's exact test, two-tailed Pearson's chi-square test. Kendall's tau-c was used for testing a trend. *P *values less than 0.05 were considered significant.

## Results

### In vivo effect of sesamin extract on the KA-induced oxidative stress

Treatment with sesamin extract (30 mg/kg) significantly increased 50% and 55.8% of plasma α-tocopherol levels from rats without and with KA treatment, compared to the baseline level, respectively. In contrast, the plasma α-tocopherol level did not change in vehicle control rats. The TBARS level of the vehicle control group was 145% of the naïve control animals. The TBARS level of the sesamin (S-30) group was 117% of the naïve control and significantly different from that of the vehicle control (V-30) (*p *= 0.017). The sesamin treatment in SE mice increased the SOD activity from 55% of the vehicle control mice to 81% of sesamin-treated mice, respectively to the normal levels. (*p *= 0.013). However at 15 mg/kg dosage of sesamin treatment, neither TBARS nor SOD activity was different from the vehicle control (V-15) (*p *> 0.05, Table 1).

**Table 1:** Effects of sesamin extract on TBARS (nmol/mg protein) and SOD (units/mg protein) in the mice with 2-h KA-induced SE

Variable	Vehicle	Sesamin extract	Naive	*P-value^a^*	*P-value^b^*		
			
	M ± SD	M ± SD	M ± SD		(V, S)	(V, N)	(S, N)
15 mg/kg							
TBARS	0.96 ± 0.22	0.85 ± 0.10	0.57 ± 0.04	0.001**	0.225	0.0002**	0.004**
SOD	4.18 ± 1.01	5.27 ± 2.01	8.79 ± 1.22	0.005**	0.686	0.003**	0.006**
30 mg/kg							
TBARS	0.86 ± 0.14	0.69 ± 0.14	0.59 ± 0.06	0.003**	0.017*	0.001**	0.126
SOD	5.17 ± 1.90	7.54 ± 2.10	9.48 ± 1.17	0.0004**	0.013*	0.0001**	0.049*

M ± SD: mean ± standard deviation. N: naïve. S: sesamin extract. V: vehicle.

^a ^One-way ANOVA test.

^b ^Least significant differences post-hoc test. *P < 0.05, **P < 0.01.

### Effect on mortality and behavior

Since the lower dosage of sesamin (15 mg/kg) did not have a good antioxidant effect, we used the higher dosage for the next experiment. The result showed that treatment with sesamin extract (30 mg/kg) on the rats with KA-induced SE decreased the mortality rate from 22% of the vehicle (V-30) group to 0% (*p *= 0.049). However, sesamin extract did not significantly attenuate the maximal seizure classes or the predominant behavioral seizure patterns as compared with the vehicle (*p *> 0.05, Tables 2 and 3). Nevertheless, there was a trend of decreasing the clonic pattern of SE by sesamin treatment that 8/23 (35%) in the vehicle (V-30) group dropped to 3/22 (14%) in the sesamin (S-30) group (Table 3).

**Table 2:** Effects of sesamin extract on the maximal seizure class (MSC) and mortality rate of the rats with 5-h KA-induced SE

Variables	Control (V-30) n (%)	Sesamin (S-30) n (%)	*P-value*
**Mortality**	5 (22)	0 (0)	0.049^a^
**MSC**			
**1-2**	2 (9)	1 (4)	0.797^b^
**3-6**	17 (74)	16 (73)	0.530^c^
**7-8**	4 (17)	5 (23)	

^a ^Fisher's exact test.

^b ^Pearson's chi-square test: all seizure classes were taken together.

^c ^Kendall's tau-c: all seizure classes were taken together.

S-30: sesamin extract (30 mg/kg). V-30: vehicle.

**Table 3:** The effect of sesamin extract on the predominant behavior patterns of the rats with 5-h KA-induced SE

Behavior Pattern	Control (V-30) n (%)	Sesamin (S-30) n (%)	*P-value*
**Immobile or Exploratory**	11 (48)	12 (54)	0.211^a^
**Masticatory**	4 (17)	7 (32)	0.323^b^
**Clonic**	8 (35)	3 (14)	

^a ^Pearson's chi-square test: all behavioral patterns were taken together.

^b ^Kendall's tau-c: all behavioral patterns were taken together.

S-30: sesamin extract (30 mg/kg). V-30: vehicle.

### In vitro protection of KA toxicity

**Figure 1:**
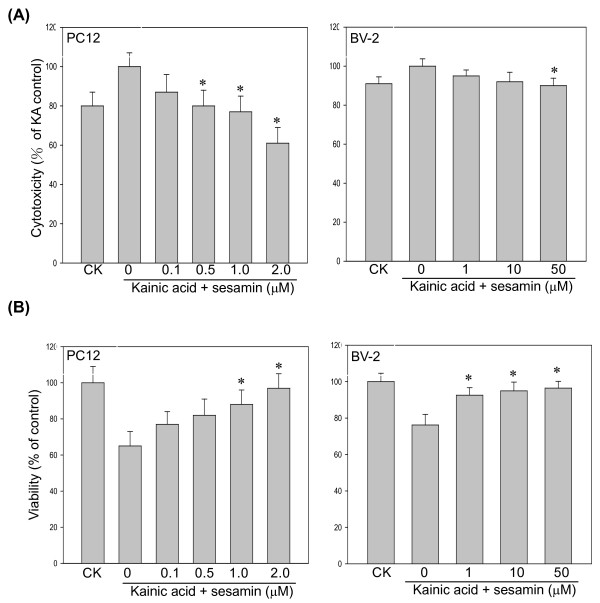
**Effect of sesamin on cell viability and cytotoxicity of kainic acid-stressed PC12 cells**. Cells were treated with KA (150 μM) alone or with various concentrations (0.1, 0.5, 1.0, 2.0 μM) of sesamin for 24 h. (A) LDH release was decreased and (B) cell viability increased by sesamin. ^* ^*P *< 0.01 as compared to KA control.

KA can induce free radicals and damage neuronal cells, therefore the cell viability and LDH released from PC12 cells were measured using MTT and LDH ELISA assays [15]. As shown in Figure 1, PC12 cells were exposed to 150 μM KA for 24 h were protected by the presence of sesamin (0.1, 0.5, 1.0, or 2.0 μM). KA-induced LDH released was reduced and the cell viability was increased by sesamin treatment. Similarly, BV-2 cells were protected by sesamin under KA stimulation (data not shown).

### KA-induced calcium release

**Figure 2:**
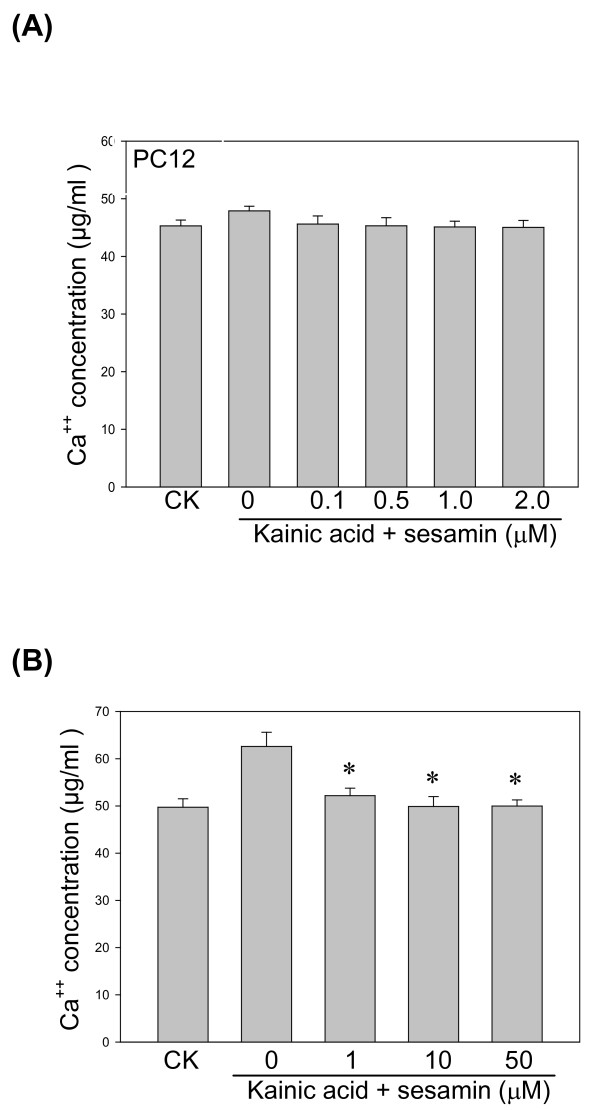
**Effect of sesamin on Ca^2+ ^generation from KA-treated PC12 cells and BV-2 cells**. Cells were treated with KA (150 μM) alone or with various sesamin concentrations (0.1-2.0 μM) of sesamin for 24 h. Treatment with sesamin effectively reduced the release of Ca^2+ ^under KA stress. ^* ^*P *< 0.01 as compared to the KA control.

KA triggers neurons membrane depolarization by the release of calcium ions [18]. Our result showed that KA induced calcium release from both PC12 and BV-2 cells in a time-dependent manner and sesamin reduced the calcium release more prominently from BV-2 cells than from PC12 cells (Figure 2).

### KA-induced ROS and lipid peroxidation

**Figure 3:**
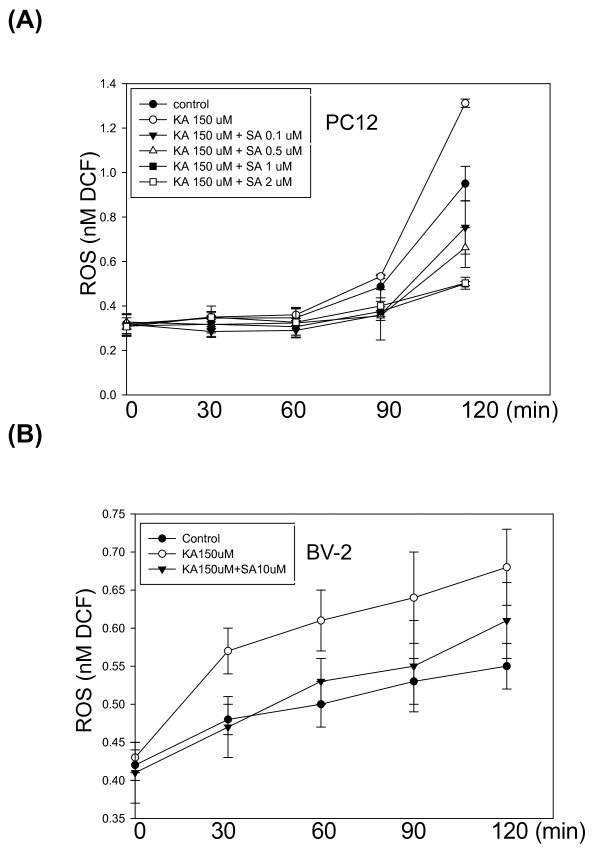
**Effect of sesamin on ROS accumulation in PC12 cells and BV-2 cells under KA stress**. Sesamin effectively reduced the ROS production from (PC12 cells induced by KA stress (150 μM) at 120-min. ^* ^*P *< 0.01 as compared to the KA control.

**Figure 4:**
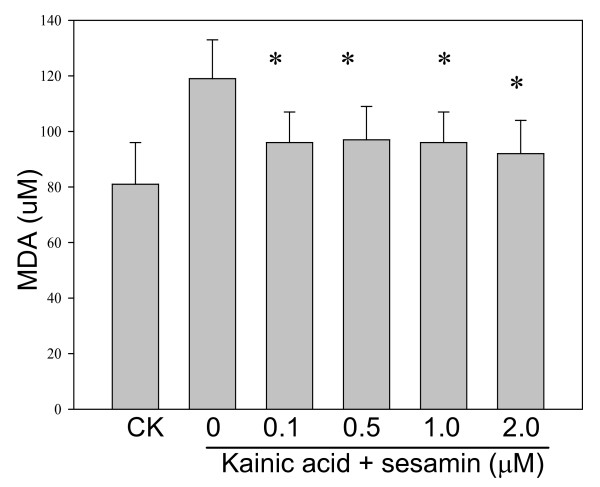
**Effect of sesamin on lipid peroxidation of PC12 cells under KA stress**. Lipid peroxidation (LPO) was determined by a LPO assay kit (Cayman Chemical, Ann Arbor, MI, USA). Malondialdehyde (MDA) of PC12 cells was induced by 24-h KA stress (150 μM) and effectively reduced by sesamin. ^* ^*P *< 0.01 as compared to the KA control.

**Figure 5:**
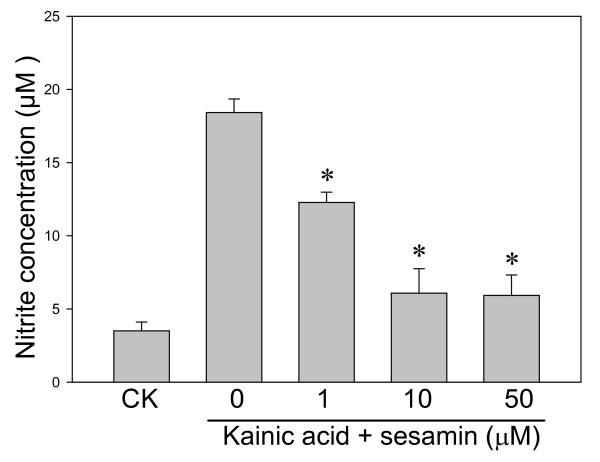
**Kainic acid-induced nitrite production**. BV-2 cells were treated with KA (150 μM) alone or with various sesamin concentrations (1-50 μM) for 24 h. KA-induced nitrite production was dose-dependently decreased the treatment with sesamin.

KA-treated PC12 cells or BV-2 cells increased DCF fluorescence nearly one-fold after 120 min as compared with the control cells. Sesamin protected cells against KA-cytotoxicity by decreasing the ROS accumulation (DCF signals) in both cells (Figure 3). Marked increase in MDA level was observed in KA-exposed cells as compared with the control cells (Figures 4-5). Treatment with sesamin significantly reduced MDA levels as compared to the KA-treated control (*p *< 0.01,).

### Caspase-3 activation

**Figure 6:**
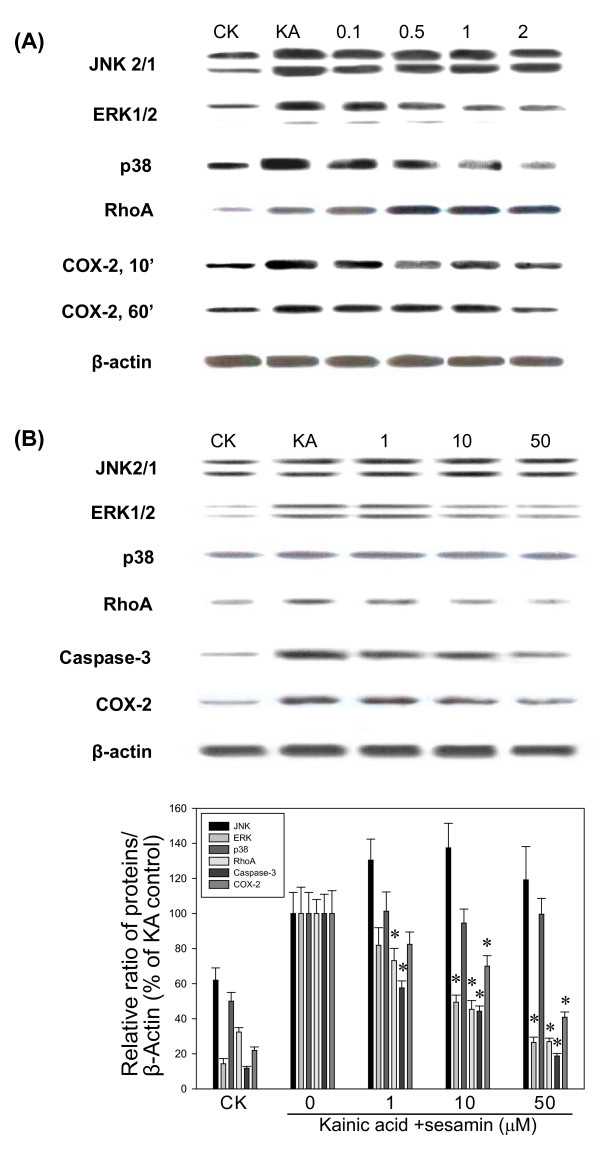
**Effect of sesamin on MAP kinases, RhoA and COX-2 activation in PC12 cells and BV-2 cells under KA stress**. The effect of sesamin (1-50 μM) on KA-activated cell signaling pathways was determined by Western blotting under KA stress, (A) PC12 cells for 10-min and 60-min and (B) BV-2 cells for 120 min. Sesamin effectively reduced the activation of COX-2, ERK, p38 MAP kinases. ^* ^*P *< 0.01 as compared to the KA control. CK: normal control.

Apoptotic signaling pathways were investigated in KA-treated PC12 cells and BV-2 cells. The results showed that KA increased Caspase-3 activation but sesamin reduced the Caspase-3 expression dose-dependently in both cells (Figure 6 and **data not shown**).

### COX-2 and MAPK activation

Seizures, inflammation and apoptosis in neuronal cells are known to be initiated with several signaling pathways such as MAPKs and Rho kinases [19-21]. Therefore, the KA induced the activation of signaling pathways were studied in PC12 cells and BV-2 cells for MAP kinases (JNK, ERK, p38), RhoA, and COX-2 (Figure 6). We evaluated the effect of sesamin on KA-induced pathways in PC12 cells at 10, 30, and 60 min and in BV-2 cells 30, 60, 120 min by Western blot. Western blot analysis revealed that sesamin (50 μM) significantly reduced ERK1/2, p38 MAPKs and COX-2 expression in both cells and RhoA expression in BV-2 cells as compared to KA controls.

### PGE_2 _production

**Figure 7:**
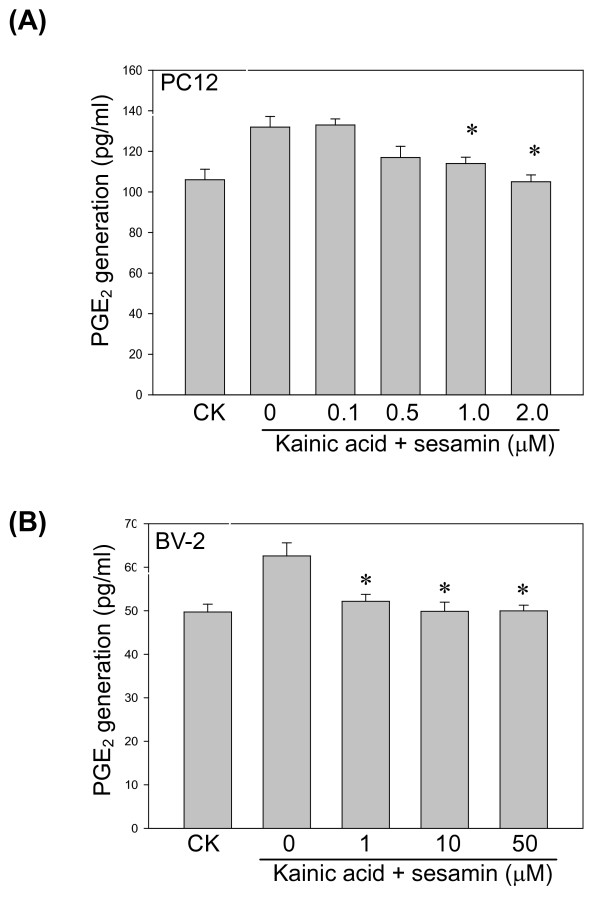
**Effect of sesamin on PGE_2 _production in PC12 cells and BV-2 cells**. Sesamin dose-dependently reduced KA-induced PGE_2 _production in (A) PC12 cells and (B) BV-2 cells. PGE_2 _concentration was determined by ELISA (R&D) assay. ^* ^*P *< 0.01 as compared to the KA control.

We further evaluated whether the KA-induced COX-2 change would affect PGE_2 _production. The result showed that sesamin reduced the PGE_2 _production in both KA-induced PC12 cells and BV-2 cells as predicted (Figure 7).

## Discussion

The present result showed that sesamin protected animals from KA-induced brain injury. MDA and mortality were significantly reduced as compared with the non-treated one. The decreased mortality in the sesamin-treated animals could also be confirmed by the sesamin effect in vitro that showed a decreased LDH release and Caspase-3 activation and increased cell viability in KA-stimulated PC12 cells.

The neuroprotective effect of sesamin on KA-induced injury was mainly due to its antioxidant effect on reducing of MDA, the product of lipid peroxidation both in vivo and in vitro. The antioxidant effect on reducing of MDA could be attributed to the increased plasma α-tocopherol level from the supplementation with sesamin extract (Table 1). This is consistent with a study that reports consumption of sesame seed powder over five weeks increases plasma α-tocopherol levels in healthy human volunteers [32]. KA administration is associated with a depletion of ATP levels and accumulation of [Ca^2+ ^]_i_. PC12 cells treated with KA for 24 h reduced cell viability dose-dependently by MTT assay. This was not caused by changes in pH or osmotic pressure because the inert mannitol at the concentration of 150 mM, did not affect cell viability (data not shown). The increase in [Ca^2+ ^]_i _may trigger Ca^2+ ^-activated free radicals formation [33]. Sesamin decreased ROS and calcium release from KA-treated PC12 cells and BV-2 cells. This agrees with the earlier reports that antioxidants can protect brain from KA-induced calcium ions and ROS release [24-26]. It is also consistent with the sesamin antioxidant effect that protects hypoxia-or H_2 _O_2 _-induced PC12 cell injury [22]. A study with defatted sesame seeds extract (30, 100 and 300 mg/kg) given twice orally at 0 h and 2 h after onset of ischemia shows the reduction of brain infarct volume dose-dependently and improves sensory-motor function [34]. Thus, suppression of KA-induced ROS and Ca^2+ ^release by sesamin was consistent with these notions. Since seizure can be triggered by the KA-induced calcium ions release, the decreased severity of seizure behavior could be partially attributed to the sesamin antioxidative effect, although it was not statistically significant due to the small sample sizes

Since microglial activation as one mechanism by which early-life seizures contribute to increased vulnerability to neurologic insults in adulthood, therapies that regulate of proinflammatory cytokines would be beneficial [35]. Present results showed that NO^. ^production in KA-stimulated BV-2 cells was dose-dependently reduced by sesamin and PGE_2 _production from both cells under KA stress was also significantly reduced. This agrees with other studies that sesamin protects PC12 cells from MPP^+ ^-induced cellular death by increasing the SOD activity and inducible nitric oxide synthase protein (iNOS) expression and microglia from reducing interleukin-6 (IL-6) mRNA levels [23]. We have reported previously that sesamin or sesamolin inhibits NO, iNOS, tumor necrosis factor-α and IL-6 production in LPS-stimulated BV-2 microglia [27]. In addition, sesamin and sesamolin also reduced LPS-activated cytokine, p38 MAPK and NF-κB activations [28].

The role of COX-2 mRNA and protein in KA-induced brain injury has been reported [36-38]. The KA-induced COX-2 expression parallels the appearance of neuronal apoptotic features [36] and involves with free radicals formation [39]. Several protease families are implicated in apoptosis, the most prominent being caspases [40]. We found that KA could affect the Caspase-3 activation in PC12 cells and BV-2 cells and sesamin could reduce both Caspase-3 expression. Since KA could induce the activation of MAP kinases (JNK, ERK, p38), RhoA and COX-2 in PC12 cells, we found that sesamin suppressed KA-induced COX-2, ERK, and p38 MAPK in PC12 cells and BV-2 cells. Our result is similar to a study of tea extract (TF3) treatment that shows TF3 reduces the gene and protein expression of COX-2 and iNOS, and NF-κB activation from cerebral ischemia-reperfusion [41]. However, KA-induced RhoA pathway in BV-2 cells but not PC12 cells was reduced by sesamin.

Our data also showed that sesamin extract had the tendency to decrease the severity of seizure behavior. A recent study indicated the lack of effectiveness of antioxidants in the kainic acid SE model [42]. However, antioxidants had significant anticonvulsant activity against pilocarpine. This discrepancy could be explained by the fact that KA is a glutamate receptor agonist while pilocarpine is a muscarinic agonist [43]. Because of the small number of animals in our study, the ameliorating effect of sesamin extract on behavioral severity was not statistically significant. Further studies are needed to confirm whether sesamin has direct effects on the seizure behavior and the related molecular mechanism in this issue. The present results are consistent with previous reports that antioxidants such as resveratrol, vitamin-E, melatonin, and lipoic acids are also protective against various animal models of SE in terms of the oxidative stress or convulsions [44-51]. Particularly, resveratrol protects against KA-induced neuronal damage [7,10,14,49]. However, in the present study the concentrations of sesamin likely to be achieved in vivo were too low to act as chain-breaking antioxidants. Only higher dose (30 mg/kg) of sesamin supplementation significantly increased plasma α-tocopherol level and reduced the TBARS level as compared with the vehicle control rats. It is more likely that sesamin acts as a pharmacological antioxidant by blocking enzymes or pathways of neuroinflammation that would produce ROS/RNS.

Stopping the seizure activity earlier is the best way to prevent SE-induced free radicals and neuronal damage. However, clinical experience shows that SE can be refractory to the commonly used medications. Therefore, intervention by antioxidants can be a potential beneficial approach in the treatment of SE.

## Conclusion

Sesamin ameliorates oxidative stress in KA-induced status epilepticus and warrants further study for the molecular mechanism.

## List of abbreviations

(COX-2) Cyclooxygenase-2, (DCF) dichlorofluorescein, (DMEM) Dulbecco's modified Eagle's medium, (FBS) fetal bovine serum, (H_2 _DCF-DA) 2',7'-dichlorodihydrofluorescein diacetate, (HBSS) Hank's balanced salt solution, (IL-6) interleukin-6, (iNOS) Inducible-nitric oxide synthase, (KA) Kainic acid, (LDH) Lactate dehydrogenase, (LPO) lipid peroxidation, (LPS) Lipopolysaccharide, (MDA) Malondialdehyde, (MAPKs) Mitogen-activated protein kinases, (MPP^+^) 1-methyl-4-phenyl-pyridine, (MTT) 3-(4,5-dimethyl-thiazol-2-yl)-2,5-diphenyl tetrazolium bromide, (NADH) nicotinamide adenine dinucleotide, (NF-κB) Nuclear factor-κB, (NO) Nitric oxide, (PBS) Phosphate-buffered saline, (PC12 cells) Pheochromacytoma, (PGE_2_) Prostaglandin E_2_, (ROS) Reactive oxygen species, (SE) Status epilepticus, (SOD) Superoxide dismutase, (TBARS) thiobarbituric acid reacting substances.

## Competing interests

The authors declare that they have no competing interests.

## Authors' contributions

PFH and CWH participated in the design and Ms editing, in all treatment procedures, data elaboration and seizure and behavior studies. MS, CL, YC participated in all treatment procedures and daily control for animal food intake, weights and SOD and TBARS assay. SPW, YFP participated in all cell-treatment procedures, participated in ROS procedures and PWY, western blot and PGE_2 _assay. KJ conceived the study and design, analyzed the data and prepared the manuscript. All authors read, discussed and approved the final manuscript.
